# Supplementary figures and images for: Identification of KIFC3 as a Colorectal Cancer Biomarker and Its Regulatory Mechanism in the Immune Microenvironment Based on Integrated Analysis of Multi-Omics Databases
Source: Biomedicines. 2025 Apr 2;13(4):859. doi: 10.3390/biomedicines13040859 (PMC12025125; doi:10.3390/biomedicines13040859)

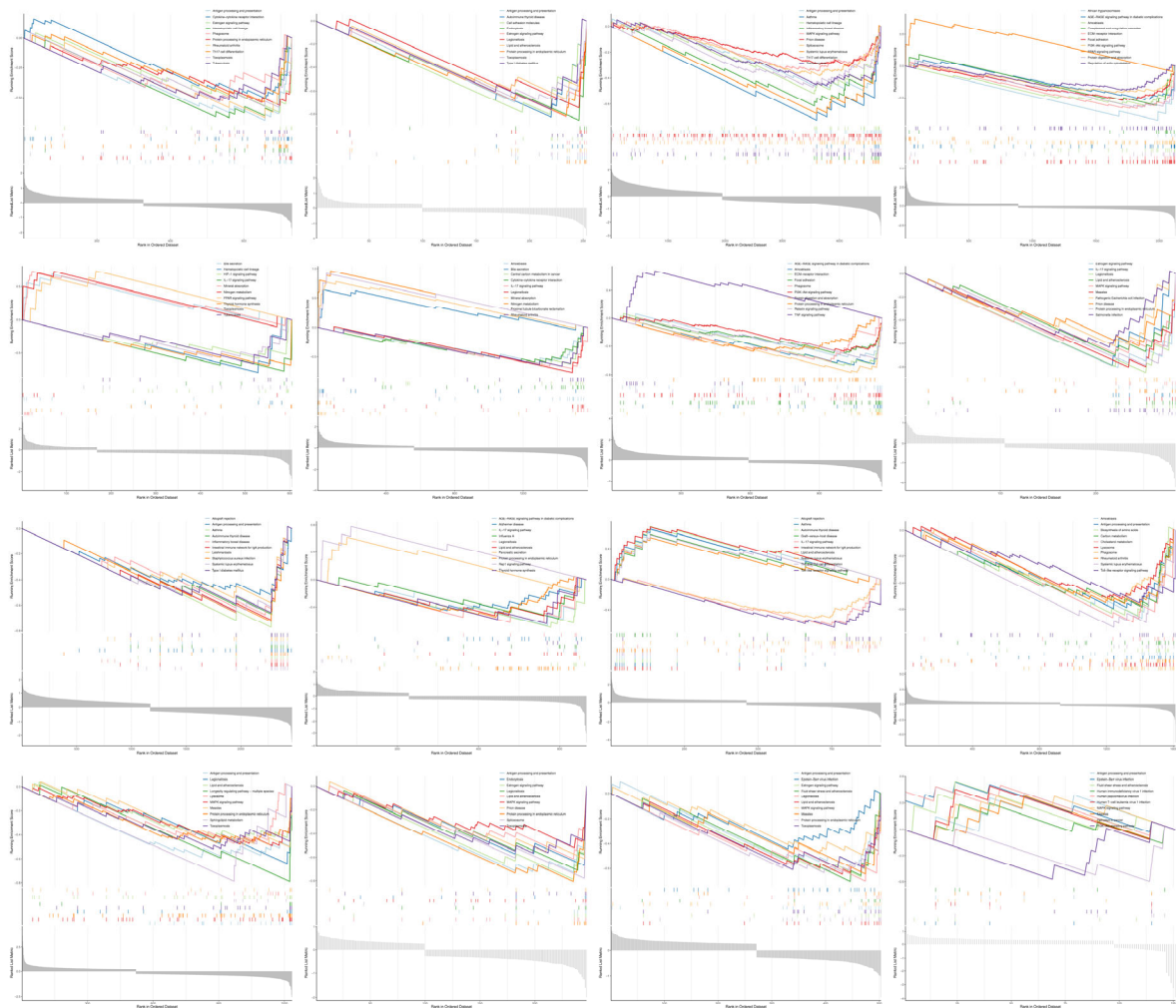

**Figure S1.** The DEGs in 16 cell clusters.

Supplement: Supplementary file 1 [file biomedicines-13-00859-s001.zip › biomedicines-3499524-SUPPL.pdf]
